# Clinical value of multi-slice 3-dimensional computed tomographic angiography in the preoperative assessment of meningioma

**DOI:** 10.3892/etm.2013.1147

**Published:** 2013-06-06

**Authors:** XIN ZHAO, RU-TONG YU, JIANG-SHAN LI, KAI XU, XIANG LI

**Affiliations:** Department of Neurosurgery, Affiliated Hospital of Xuzhou Medical College, Xuzhou, Jiangsu 221000, P.R. China

**Keywords:** 3-dimensional computed tomographic angiography, meningiomas, surgery

## Abstract

The aim of this study was to evaluate the clinical value of multislice 3-dimensional computed tomographic angiography (3D-CTA) in the preoperative assessment of meningiomas. A total of 331 cases with meningiomas confirmed by CT and MRI were examined using 3D-CTA. The locations of the tumors were observed to be as follows: parasagittal and falcine in 125 cases, sphenoidal in 39 cases, in the olfactory groove in 19 cases, tentorial in 21 cases, parasellar in 33 cases, petroclival in 29 cases, intraventricular in 7 cases and on the convexity of the brain in 58 cases. The reconstructed images were processed by shaded volume rendering, maximum intensity projection and color-shaded surface display. The 3D-CTA images were used to imitate the surgical approach. Surgery was performed according to the information provided in the 3D-CTA images. 3D-CTA provided clear 3D images of the meningioma and the relationship with the adjacent vessels and the skull base, and demonstrated the optimal surgical approach for removing the neoplasm. The results of 3D-CTA corresponded extremely well with the surgical observations. 3D-CTA is able to provide 3D images of the meningioma, adjacent vessels and the bones in the skull base. Furthermore, 3D-CTA supplies information vital in the selection of the optimal surgical approach and information that aids the management of the sinus during the surgery. 3D-CTA is of great value in the preoperative evaluation of meningiomas.

## Introduction

Meningiomas are the most frequent type of intracranial tumors (1,2). However, treatments for meningiomas using microsurgical manipulation are highly challenging due to several factors, such as the location of the meningioma, complicated surrounding vascular networks including the deep veins and supplying arteries, and the anatomical obstacles of the parasagittal and falx (3,4). It is important for the neurosurgeon to obtain the most precise information concerning the degree of tumor involvement of critical vascular structures prior to surgery (5). The advent of spiral computed tomography has led to the development of 3-dimensional computed tomographic angiography (3D-CTA) (6). 3D-CTA provides a noninvasive and rapid diagnosis of intracranial aneurysms (7–9). 3D-CTA is a convenient technique that provides a 3D visual reconstruction of the tumor and its blood supply. However, few studies concerning the 3D-CTA imaging of meningioma have been reported. To investigate the role of 3D-CTA in the preoperative evaluation of meningioma, we have developed a protocol for performing 3D-CTA in cranial meningioma. The reliability of 3D-CTA in the detection and evaluation of meningioma is compared with that of microsurgical findings. The primary purpose of this study was to objectively compare the anatomical information provided by 3D-CTA with the results obtained by surgery.

## Materials and methods

### Patients

Between October 2001 and May 2012, a total of 331 patients with meningiomas confirmed by CT and MRI were examined by 3D-CTA. The patients comprised 116 men and 215 women, ranging in age from 34 to 78 years (mean 45.9 years). The locations of the tumors were observed to be parasagittal and falcine in 125 cases, sphenoidal in 39 cases, in the olfactory groove in 19 cases, tentorial in 21 cases, parasellar in 33 cases, petroclival in 29 cases, intraventricular in 7 cases and on the convexity of the brain in 58 cases. Informed consent was obtained from patients and the study was approved by the ethics committee of Xuzhou Medical College.

### Methods

3D-CTA was performed on the patients with the use of a General Electric LightSpeed Plus CT scanner (General Electric, Milwaukee, WI, USA). An intravenous catheter was inserted in the antecubital vein. Iobitridol (1.5 ml/kg) was prepared for administration by a power injector (Medrad, Indianola, PA, USA) at a rate of 3.0 ml per second. The scans were prescribed starting at 40 sec after the injection. These images were further reconstructed with the General Electric Advantage Windows 3-D workstation (General Electric). The reconstructed images were then processed at the workstation into color-shaded surface display (SSD), maximum intensity projection (MIP) and shaded volume rendering (SVR) images. This was performed by the scanner technician, with either a neurosurgeon or neuroradiologist to provide editing assistance. We rotated the 3D-CTA images from every point of view in order to display the meningioma and the relationship of the cranial bone and vessels surrounding the tumor. 3D-CTA provides imaging features to suggest the diagnosis of meningioma and to delineate the cortical and vascular anatomy for preoperative planning.

## Results

**Figure 1.**
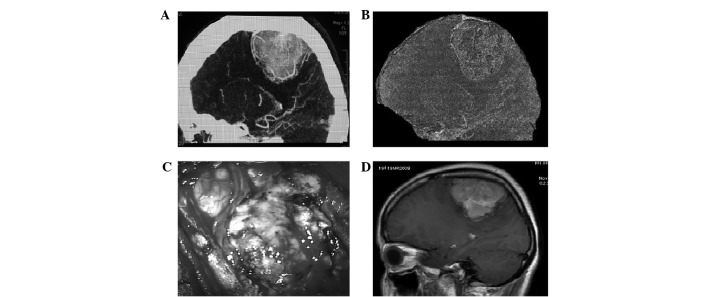
Case 1: Parasagittal and falcine meningioma. (A) 3D-CTA clearly demonstrated the relationship between the tumor, drain vein and cranium. (B) 3D-CTA clearly demonstrated the relationship between the tumor and drain vein. (C) The observation during the operation was consistent with the 3D-CTA image. The tumor was removed with the vessels intact. (D) The regular MRI failed to demonstrate the relationship between the tumor and drain vein. 3D-CTA, 3-dimensional computed tomographic angiography.

**Figure 2.**
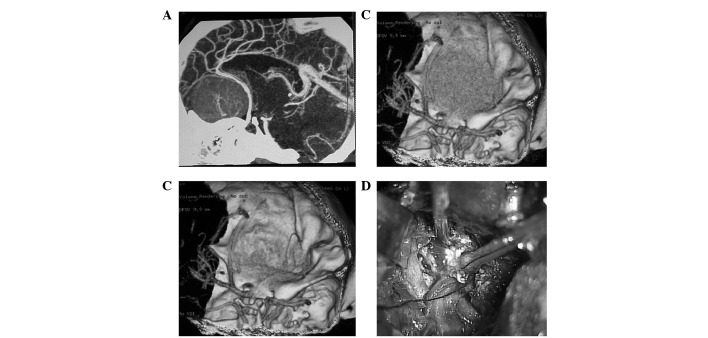
Case 2: Olfactory groove meningioma. (A) 3D-CTA clearly demonstrated the relationship between the tumor and ACA. (B) 3D-CTA clearly demonstrate the relationship between the tumor and ICA. (C) 3D-CTA clearly demonstrated the relationship between the tumor, drain vein and cranium. (D) The observation during the operation was consistent with the 3D-CTA image. ACA, anterior cerebral artery; ICA, internal carotid artery; 3D-CTA, 3-dimensional computed tomographic angiography.

Among the 125 patients with parasagittal and falcine meningiomas, 3D-CTA demonstrated that the sagittal venous sinuses were partially occluded in 109 cases (Fig. 1). The anterior third of the sagittal sinus was completely occluded in 16 cases. 3D-CTA demonstrated the effect of tumors on major cerebral arteries. Among the 39 patients with sphenoidal ridge meningioma, displacement of the internal carotid artery (ICA), anterior cerebral artery (ACA) and middle cerebral artery (MCA) were shown in 32 cases, and encasement in 7 cases. Among the 19 patients with olfactory groove meningioma, the ACA, MCA and their proximal branches were displaced in 16 patients (Fig. 2), and encased in 3 patients. Among the 33 patients with parasellar meningioma, displacement of the ICA, ACA and MCA were shown in 29 cases, with encasement in 4 cases. 3D-CTA demonstrated the relationship of the tumor, bone, clivus and basilar artery clearly among the 29 patients with petroclival meningiomas. The relationship of the transverse sinus and tumor were demonstrated clearly in 21 cases of tentorial meningioma. The relationship of the cortical vasculature and the tumor were demonstrated clearly in 27 cases of intraventricular and convexity meningioma. The 3D-CTA images corresponded well to the surgical findings.

## Discussion

Meningiomas are the most frequent group of intracranial tumors, accounting for approximately one-third of all primary brain tumors (10). The major problem in management of meningioma is the increased vascularity of the tumor (1). The management of intraoperative bleeding during the removal of large meningiomas is crucial for safe and efficient surgery (11–13). It is important to the neurosurgeon to obtain the most precise information concerning the degree of tumor involvement with critical vascular structures.

3D-CTA performed with a spiral CT scanner has been used for intracranial aneurysm detection (6–9). 3D-CTA offers a tremendous ability to provide anatomical information regarding aneurysms. 3D-CTA may provide sufficient preoperative cranial vascular information about the meningioma. In the current study, 3D-CTA not only showed the tumors stained by iobitridol, but also clearly depicted the arteries and veins surrounding the neoplasms. The division of the sinus into thirds proves useful in clarifying technical considerations that concern the operative management of the sagittal sinus itself when it is involved with the tumor (14). The middle third of the sagittal sinus lies adjacent to the paracentral lobule and the motor and sensory cortex for the feet and lower legs. This area is drained by a cortical vein, or group of veins, which should be preserved if it is patent at the time of microsurgery (15). Among the 125 patients with parasagittal and falcine meningioma, 3D-CTA demonstrated that the sagittal venous sinus was partially occluded in 109 cases (Fig. 1). The anterior third of the sagittal sinus was completely occluded in 16 cases. 3D-CTA provides imaging features to suggest the diagnosis of meningioma and to delineate the cortical and vascular anatomy for microsurgical planning. 3D-CTA not only demonstrated the relationship of the tumor and sagittal sinus but also provided the location of major cortical draining veins. 3D-CTA is useful in clarifying technical considerations that concern the surgery by demonstrating the relationship between the tumor and sagittal sinus (16). According to the images obtained by 3D-CTA, great care was taken during the dissection of the posterior portion of the capsule to preserve the cortical veins, and complete dissection of the tumor was achieved. The following should also be considered prior to surgery on parasagittal and falcine meningiomas: i) the patency of the sagittal venous sinus, including partial or complete occlusion by tumor; ii) the relationship of major cortical draining veins to the tumor (15); iii) the relationship of the branches from the internal carotid artery to the tumor; and iv) the location of the vessels surrounding the tumor relative to the planned craniotomy exposure. 3D-CTA provides vascular information critical for microsurgical planning; it provides data regarding the feeding vessels to the tumor, tumor staining and vascular shift. In the current study, these results were observed to be consistent with the findings during surgery, which demonstrated that 3D-CTA is a valuable tool for analyzing tumor blood supply and vascular shift preoperatively. 3D-CTA is a useful technique for detecting the feeding vessels of the tumor during tumor removal and for reducing intraoperative blood loss and operative time. On the basis of 3D-CTA, we conclude that if the sinus is partially invaded, it may be opened to obtain as complete a resection as possible and to attempt to preserve the patency of the sinus. If the sinus is obstructed, the portion of the sinus involved may be resected completely. In both situations, extreme care is vital for the preservation of cortical veins, which may offer important collateral drainage (14–16). For those patients with parasagittal and falcine meningiomas, the anatomical information available from 3D-CTA has been of substantial value. In our experience, the images available from 3D-CTA have been useful for the sophisticated preoperative planning of the meningioma. 3D-CTA clearly shows the relationship between vessels and the tumor, and also enables the vessels to be protected from damage during surgery (17).

In addition to preoperative information, the application of surgical approach simulation is useful in choosing an approach for moving the meningioma. Usually, a rim of cerebral cortex or arachnoid separates the main trunk of the arteries from the tumor, although occasionally the artery may be engulfed by the tumor (18). In many cases, alternative and reasonable surgical approaches are available for dealing with the meningioma. Olfactory groove meningiomas may be accessed via a subfrontal or pterional approach (19). 3D-CTA is able to identify the position of the ICA, MCA and other vessels, and is able to define the ICA when encased or displaced by the tumor. 3D-CTA depicts the relationship between skull base meningiomas and neighboring bony and vascular structures clearly. It is extremely useful to know the relationship of the ICA to the tumor and the relationship of the tumor to the cranial bone while planning a patient’s microsurgical approach (18,19). Among the 19 patients with olfactory groove meningioma, the anterior, middle cerebral arteries and their proximal branches were displaced in 16 patients whose tumors were moved through a subfrontal approach, and the ICA was encased in 3 patients whose tumors were moved through a pterional approach (Fig. 2). The findings of this study suggest that preoperative 3D-CTA may greatly aid in the understanding of the anatomical relationship between the surrounding venous system, the tumor and its blood supply. 3D-CTA is able to show clearly the relationship of the petroclival meningioma attached to the basilar artery and surrounding structures (20). Thus, preoperative evaluations using 3D-CTA aided the decisions regarding the microsurgical approach in the 29 patients with petroclival meningiomas. The tumor-bone relationships and tumor-vasculature relationships from 3D-CTA are important to the preoperative assessment. With 3D-CTA, we were able to obtain clear images revealing the relationships between the sphenoidal ridge meningioma and surrounding structures. The microsurgical findings indicated that 3D-CTA provided useful information concerning the relationships of parasellar meningioma, bone and blood vessels.

In summary, 3D-CTA is a quick, reliable and noninvasive diagnostic tool for meningioma, 3D-CTA depicts the relationship between skull base meningiomas and neighboring bony and vascular structures clearly. The anatomical information available from 3D-CTA is useful for surgical planning. Useful information concerning the cortical venous drainage, sinus patency and displacement of major arteries in patients with meningioma may be obtained by 3D-CTA. We suggest that 3D-CTA plays an important role in the preoperative evaluation of meningiomas.
